# EPIGENE: genome-wide transcription unit annotation using a multivariate probabilistic model of histone modifications

**DOI:** 10.1186/s13072-020-00341-z

**Published:** 2020-04-07

**Authors:** Anshupa Sahu, Na Li, Ilona Dunkel, Ho-Ryun Chung

**Affiliations:** 1 Institute for Medical Bioinformatics and Biostatistics, Philipps University of Marburg, 35037 Marburg, Germany; 2 Otto-Warburg-Laboratory, Max Planck Institute for Molecular Genetics, 14195 Berlin, Germany; 3 Guangzhou Institute of Pediatrics, Guangzhou Women and Children’s Medical Center, Guangzhou, 510623 China

**Keywords:** Transcription, Epigenetics, Histone modifications, Hidden Markov model, Transcript identification

## Abstract

**Background:**

Understanding the transcriptome is critical for explaining the functional as well as regulatory roles of genomic regions. Current methods for the identification of transcription units (TUs) use RNA-seq that, however, require large quantities of mRNA rendering the identification of inherently unstable TUs, e.g. miRNA precursors, difficult. This problem can be alleviated by chromatin-based approaches due to a correlation between histone modifications and transcription.

**Results:**

Here, we introduce EPIGENE, a novel chromatin segmentation method for the identification of active TUs using transcription-associated histone modifications. Unlike the existing chromatin segmentation approaches, EPIGENE uses a constrained, semi-supervised multivariate hidden Markov model (HMM) that models the observed combination of histone modifications using a product of independent Bernoulli random variables, to identify active TUs. Our results show that EPIGENE can identify genome-wide TUs in an unbiased manner. EPIGENE-predicted TUs show an enrichment of RNA Polymerase II at the transcription start site and in gene body indicating that they are indeed transcribed. Comprehensive validation using existing annotations revealed that 93% of EPIGENE TUs can be explained by existing gene annotations and 5% of EPIGENE TUs in HepG2 can be explained by microRNA annotations. EPIGENE outperformed the existing RNA-seq-based approaches in TU prediction precision across human cell lines. Finally, we identified 232 novel TUs in K562 and 43 novel cell-specific TUs all of which were supported by RNA Polymerase II ChIP-seq and Nascent RNA-seq data.

**Conclusion:**

We demonstrate the applicability of EPIGENE to identify genome-wide active TUs and to provide valuable information about unannotated TUs. EPIGENE is an open-source method and is freely available at: https://github.com/imbbLab/EPIGENE.

## Background

Transcription units (TUs) represent the transcribed regions of the genome which generate protein-coding genes as well as regulatory non-coding RNAs like microRNAs. Accurate identification of TUs is important to better understand the transcriptomic landscape of the genome. With the rapid development of low-cost high-throughput sequencing technologies, RNA sequencing (RNA-seq) has become the major tool for genome-wide TU identification. Hence, popular TU prediction tools such as AUGUSTUS [1], Cufflinks [2], StringTie [3], Oases [4] use RNA-seq data. Though RNA-seq-based TU prediction can be considered the state-of-the-art method to annotate the genome, its main drawback lies in its dependence on relatively high quantities of target RNAs. This is problematic for accurate identification of inherently unstable TUs like primary miRNA, etc. Recent studies have reported the presence of large number of TUs that are rapidly degraded [5–7], some of which have been associated with diseases like HIV [8], cancer [9–11], Alzheimer’s disease [12, 13], etc. While some unstable microRNA precursors have been identified by nascent transcription approaches like GRO-seq [14], PRO-seq [15], NET-seq [16], TT-seq [17], these approaches, however, are laborious, time-consuming, limited to cell cultures, and require high amount of input material (range of 10^7^ cells) [18–20]. In addition, most of these techniques were designed to answer very specific questions about RNA Polymerase II transcription and hence identify very specific stages of transcription such as transcription start site (TSS), RNA Polymerase II C-terminal domain modification, etc. [20]. These shortcomings of existing approaches can be alleviated with chromatin-based approaches [21, 22], due to the association between histone modifications and transcription.

Eukaryotic DNA is tightly packaged into macromolecular complex called chromatin, which consists of repeating units of 147 DNA base pairs (bp) wrapped around an octamer of four histones H2A, H2B, H3, and H4 called the nucleosome. Post-translational modifications (PTM) to histones in the form of acetylation, methylation, phosphorylation, and ubiquitination, play an important role in the transcriptional process. These PTMs are added, read, and removed by so-called writers, readers, and erasers, respectively. In this way nucleosomes serve as signalling platforms [23] that enable the localized activity of chromatin signalling networks partaking in transcription and other chromatin-related processes [24]. Indeed, it has been shown that histone modifications are correlated to the transcriptional status of chromatin [25, 26]. For example, H3K4me3 and H3K36me3 are positively correlated with transcription initiation [27, 28] and elongation [29] and are considered as transcription activation marks, whereas H3K9me3 and H3K27me3 are considered as repressive marks as they are commonly found in repressed regions [27, 30]. Therefore, it is reasonable to assume that histone modifications profiles can be used to identify cell type-specific TUs. Given a deluge of cell type-specific epigenome data available through many consortia, such as ENCODE [31], NIH Roadmap Epigenomics [32], DEEP [33], Blueprint [34], CEEHRC [35], and IHEC [36], a highly robust TU annotation pipeline based on epigenome markers becomes feasible.

Currently many computational approaches such as ChromHMM [37], EpicSeg [38], chroModule [39], GenoSTAN [40], etc., are available that use histone modifications as input to provide genome-wide chromatin annotation. These chromatin segmentation approaches use a variety of mathematical models with the most prominent one being hidden Markov models (HMM). These HMMs model the observed combination of histone modifications emitted by a sequence of hidden chromatin states according to emission probabilities. Moreover, the hidden chromatin states are linked by transition probabilities that introduce correlations in the observed histone modifications.

Based on the training, these HMMs can be classified as: (a) unsupervised methods that do not include prior biological information and require users to interpret and annotate the learned states based on existing knowledge about functional genomics (e.g. ChromHMM, EpicSeg, and GenoSTAN) and (b) supervised methods, that rely on a set of positive samples for training (e.g. chroModule). Although these approaches annotate genome modules such as promoter, enhancer, transcribed regions, etc., they fail to identify active TUs as they do not constrain the chromatin state sequence to begin with a transcription start site (TSS) and end with a transcription termination site (TTS).

To address these shortcomings, we developed a semi-supervised HMM, EPIGENE (EPIgenomic GENE), which is trained on the combinatorial pattern of IHEC class 1 epigenomes (H3K27ac, H3K4me1, H3K4me3, H3K36me3, H3K27me3, and H3K9me3) to infer hidden “transcription unit states”. The emission probabilities represent the probability of a histone modification occurring in a TU state and the transition probabilities capture the topology of TU states. In addition to the TU states, the HMM also includes background states. The transcription start site (TSS), exons (first, internal, and last exon), introns (first, internal, and last intron) and transcription termination site (TTS) are referred to as the TU states. The emission probabilities of these states as well as the transition probabilities between them are learned from the structure of TUs given by an existing transcript annotation. The transition and emission probabilities of the background states, the transition probabilities from and to TSS and TTS states, and the transition probabilities between TSS and TTS states are learned in an unsupervised manner from the data.

In the forthcoming sections, we describe the EPIGENE approach, validate the predicted EPIGENE TUs with existing annotations, RNA-seq, and ChIP-seq evidence, compare the performance of EPIGENE to existing chromatin segmentation and RNA-seq-based methods within and across cell lines, and show that EPIGENE outperforms state-of-the-art RNA-seq and chromatin segmentation approaches in prediction resolution and precision. In summary, EPIGENE yields predictions with a high resolution and provides a pre-trained robust model that can be applied across cell lines.

## Results and discussion

### Schematic overview of EPIGENE

**Fig. 1:**
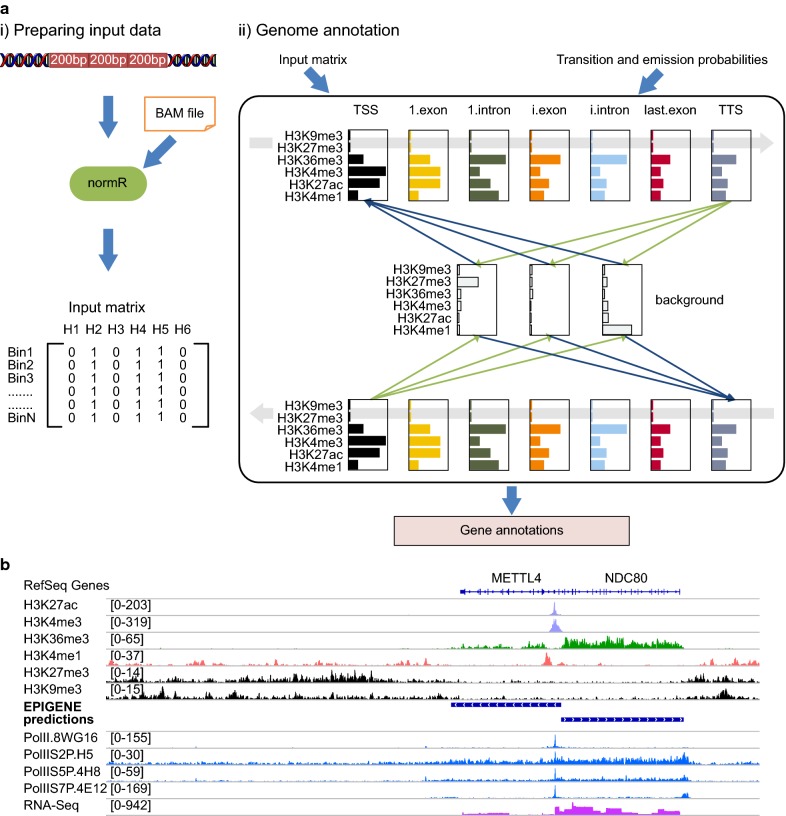
**a** Schematic overview of EPIGENE framework. **b** An example of EPIGENE prediction. EPIGENE predictions of METTL4 and NC80 gene show an enrichment of H3K27ac and H3K4me3 at TSS (tracks shown in light violet), H3K36me3 in gene body (tracks shown in green), enhancer mark H3K4me1 few bps upstream or downstream of TSS (tracks shown in pink), RNA Polymerase II in TSS and gene body (tracks shown in blue). The predictions also show an absence of repression marks H3K27me3 and H3K9me3 (tracks shown in black). The corresponding RNA-seq evidence in this genomic region can be seen in the lower-most track (track shown in dark pink)

EPIGENE uses a multivariate HMM, which allows the probabilistic modelling of the combinatorial presence and absence of multiple IHEC class 1 histone modifications. It receives a list of aligned ChIP and control reads for each histone modification, which is subsequently converted into presence or absence calls across the genome using normR (see “Binarization of ChIP-seq profiles” section; Fig. 1a (i)). By default, TU states were analysed at 200-bp non-overlapping intervals called bins. The HMM comprises 14 TU states and 3 background states where each TU state captures individual elements of a gene (i.e. TSS, exons, introns, and TTS). The TU state sequence was duplicated, running from TSS to TTS and from TTS to TSS, allowing identification of TUs on the forward and reverse strand, respectively (see Fig. 1a (ii)). The transition probabilities between the TU states were trained in a supervised manner using GENCODE annotations [41] and their emission probabilities were trained on a highly confident set of GENCODE transcripts [41] that showed an enrichment for RNA Polymerase II in K562 cell line (see “Training the model parameters” section). The transition and emission probabilities of background states, the transition probabilities from or to either the TSS or TTS state, and the transition probabilities between TSS and TTS states were trained in an unsupervised manner (see “Training the model parameters” section). The HMM outputs a vector where each bin is assigned to a TU state or to one of the three background states. This vector is then further refined to obtain active TUs (see Fig. 1b).

### Validation with existing gene annotations and RNA-seq

**Fig. 2:**
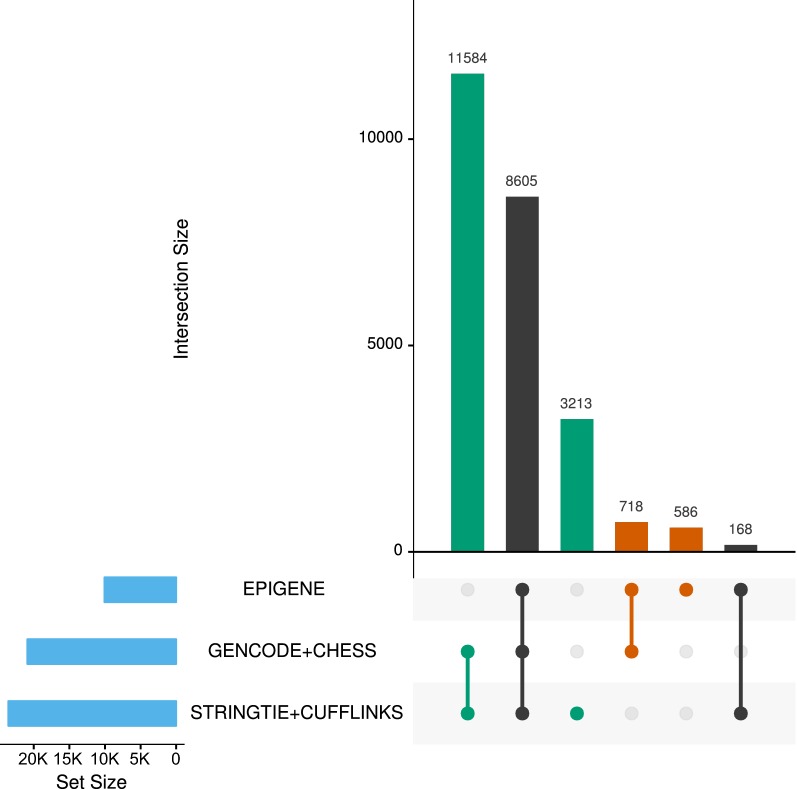
Overlap of EPIGENE predictions with existing gene annotations and RNA-seq-based predictions

We validated the predicted TUs using existing gene annotations and RNA-seq evidence. For this, we combined the EPIGENE predictions (24,571 TUs) and RNA-seq predictions that were obtained from Cufflinks (32,079 TUs) and StringTie (101,656 TUs; Additional file 1: Tables S2–S4 for summary statistics) to generate a consensus TU set. This consensus TU set contains 24,874 TUs, which were then overlaid with GENCODE and CHESS gene annotation [41, 42] (Fig. 2). We found that 93% of EPIGENE TUs can be explained by existing gene annotations. We identified 14,797 (11,584: annotated, 3213: unannotated) RNA-seq-exclusive TUs and 1304 (718: annotated, 586: unannotated) EPIGENE-exclusive TUs. Additional integration of RNA Polymerase II ChIP and Nascent RNA-seq data revealed that 40% (232 out of 586 TUs) of EPIGENE unannotated TUs and 35% (1120 out of 3213 TUs) of RNA-seq unannotated TUs showed enrichment of RNA Polymerase II ChIP, TT-seq, and GRO-seq evidence. Also, 88.4% (518 out of 586 TUs) could be validated by either RNA Polymerase II ChIP or Nascent RNA-seq. Additional details about RNA Polymerase II ChIP and Nascent RNA-seq enrichment in the consensus TU set can be seen in Additional file 2: Table S5.

### Histone modifications and RNA Polymerase II occupancy

**Fig. 3:**
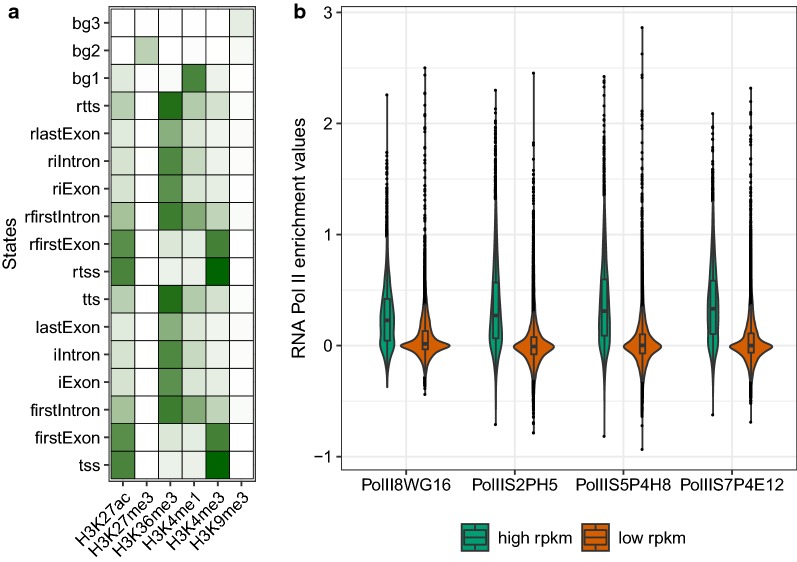
Correctness of EPIGENE predictions. **a** EPIGENE-estimated parameters for K562 using 17 chromatin states, ranging from 0 (white) to 1 (dark green). **b** Distribution of RNA Polymerase II enrichment score in EPIGENE predictions. The EPIGENE predictions are classified as: high RPKM (RPKM ≥ upper quartile) and low RPKM (RPKM < upper quartile) based on RNA-seq evidence in predicted transcripts

The correctness of predicted TUs was estimated in K562 cell line, due to the availability of matched RNA Polymerase II and RNA-seq profiles. We predicted 24,571 TUs in K562 majority of which showed typical gene characteristics, with high enrichment of H3K27ac, H3K4me3 and H3K36me3 in TSS and gene bodies (Fig. 3a).

It is known that eukaryotic transcription is regulated by phosphorylation of RNA Polymerase II carboxy-terminal domain at serine 2, 5 and 7. The phosphorylation signal for serine 5 and 7 is strong at promoter region, whereas signal for serine 2 and 5 is strong at actively transcribed regions [43]. Genome-wide RNA Polymerase II profile for K562 cell line was obtained using four antibodies (see “Library preparation of RNA polymerase II ChIP-seq” section) that capture RNA Polymerase II signal at transcription initiation and gene bodies. The enrichment of RNA Polymerase II in predicted TUs was computed using normR [44] (see “Binarization of ChIP-seq profiles” section). The predicted TUs were classified as having high or low RPKM based on mRNA levels (threshold = upper quartile). Figure 3b shows the distribution of RNA Polymerase II enrichment in both the classes of predicted TUs. We observed that a significant proportion of predicted TUs (78%) showed an enrichment of RNA Polymerase II and thus were likely to be true positives. We also came across 24 unannotated TUs that showed an enrichment of RNA Polymerase II (enrichment score above 0.5), but had reduced or no RNA-seq evidence.

### Comparison with RNA-seq-based approaches

**Fig. 4:**
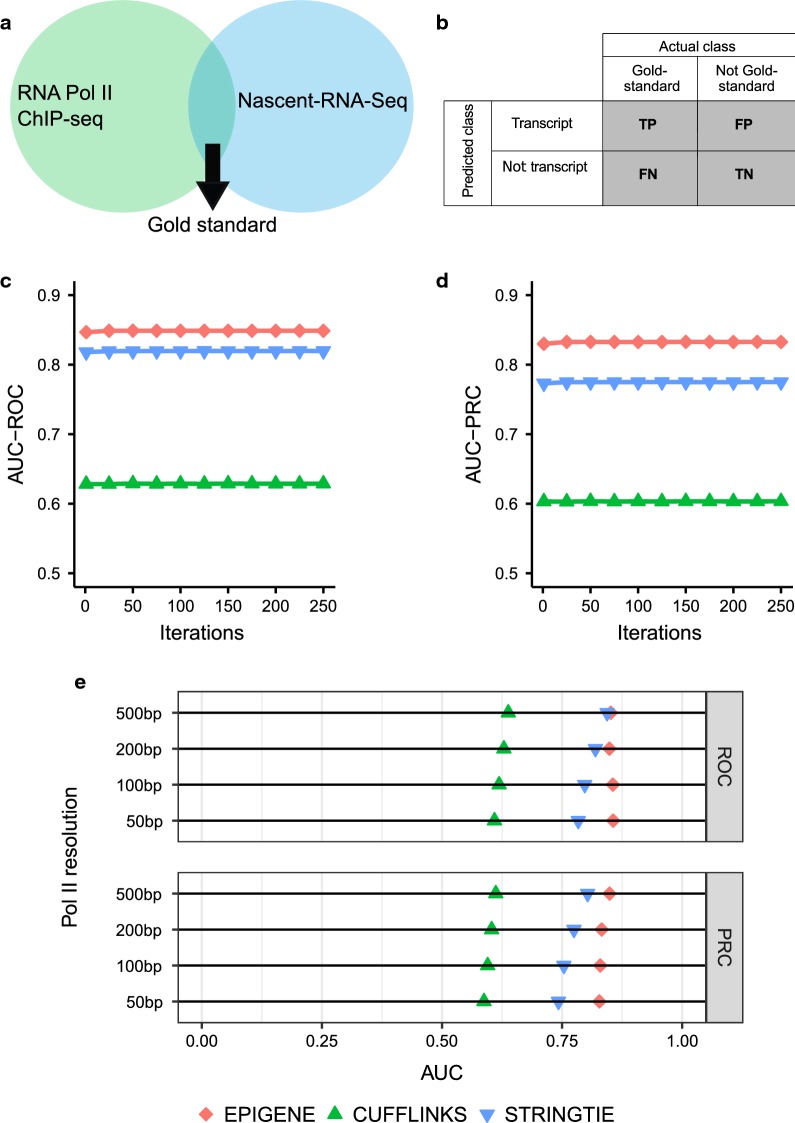
Performance of EPIGENE compared to existing RNA-seq-based transcription unit prediction methods: Cufflinks and StringTie. **a** Set of gold standard regions obtained by combining RNA Polymerase II ChIP-seq and Nascent RNA-seq profiles. **b** Contingency matrix used for method comparison. **c** Receiver-operating characteristic curve. **d** Precision–recall curve. **e** Area under ROC and PRC curve for varying RNA Polymerase II resolution for EPIGENE, Cufflinks and StringTie

Currently, there is no gold standard set of true TUs. However, there is a plethora of experimental approaches for studying RNA Polymerase II transcription. In order to perform an unbiased comparison, we integrated RNA Polymerase II data from ChIP-seq and Nascent RNA-seq techniques. For individual cell lines, we defined a set of gold standard regions based on RNA Polymerase II ChIP-seq and Nascent RNA-seq evidence (see Fig. 4a). We compared the performance of EPIGENE with two existing RNA-seq based transcript prediction approaches, Cufflinks and StringTie, both of which are known to predict novel TUs in addition to annotated TUs. The method comparison was performed in two stages: within-cell type and cross-cell type comparison using RNA Polymerase II ChIP-seq and Nascent RNA-seq enrichment as performance indicator (see “Performance evaluation” section, Fig. 4b).

#### Within-cell type comparison

For this comparison, we used the ChIP-seq profile of RNA Polymerase II in K562 cell line and the pre-existing nascent RNA TUs reported by Schwalb et al. [17] as performance indicator (see “Binarization of Nascent RNA-seq profiles” and “Performance evaluation” sections). The nascent RNA TUs have been reported to show an enrichment of TT-seq and GRO-seq [17]. The ChIP-seq profiles of RNA Polymerase II were obtained using PolIIS5P4H8 antibody because it can enrich RNA Polymerase II both at the TSS and in actively transcribed regions.

We performed the method comparison at 200-bp resolution and found that EPIGENE reports in both the precision–recall curve (PRC) and the receiver-operating characteristic (ROC) curves a higher AUC (PRC: 0.83, ROC: 0.85; Fig. 4c, d) compared to Cufflinks (PRC: 0.60, ROC: 0.63) and StringTie (PRC: 0.77, ROC: 0.82). We repeated this analysis for three different resolutions (50, 100, and 500 bp) and the corresponding AUC values are in Fig. 4e. Cufflinks achieved a lower AUC compared to StringTie and EPIGENE, which is likely due to the usage of the RABT assembler which results in large number of false positives [45].

StringTie reported a lower AUC than EPIGENE for varying RNA Polymerase II resolutions. We examined the precision, sensitivity, and specificity values for EPIGENE, Cufflinks, and StringTie and found that the lower AUC for RNA-seq-based methods was due to spurious read mappings of RNA-seq that results in higher false positives in StringTie and Cufflinks. Additional file 1: Figure S1 shows an example of Cufflinks and StringTie TU that was identified due to spurious read mapping. This TU exactly overlaps with a repetitive sequence that occurs in four chromosomes (chromosome 1, 5, 6, X).

#### Cross-cell type comparison

For this comparison, we used three different datasets provided by the GEO database [46], ENCODE [31], and DEEP [33] consortium:

1. IMR90: lung fibroblast cells with 6 histone modifications obtained from Lister et al. [47], one RNA Polymerase II obtained from Dunham et al. [48], two control experiments (one each for RNA Polymerase II [48] and histone modifications [47]), one RNA-seq obtained from Dunham et al. [48] and one GRO-seq profile obtained from Jin et al. [49],
2. HepG2 replicate 1 and HepG2 replicate 2: hepatocellular carcinoma with 6 histone modifications, one control experiment and one RNA-seq obtained from Salhab et al. [50] where two replicates per histone modification and RNA-seq were available, RNA Polymerase II ChIP and control experiments obtained from Dunham et al. [48] and one GRO-seq obtained from Bouvy-Liivrand et al. [51].

**Fig. 5:**
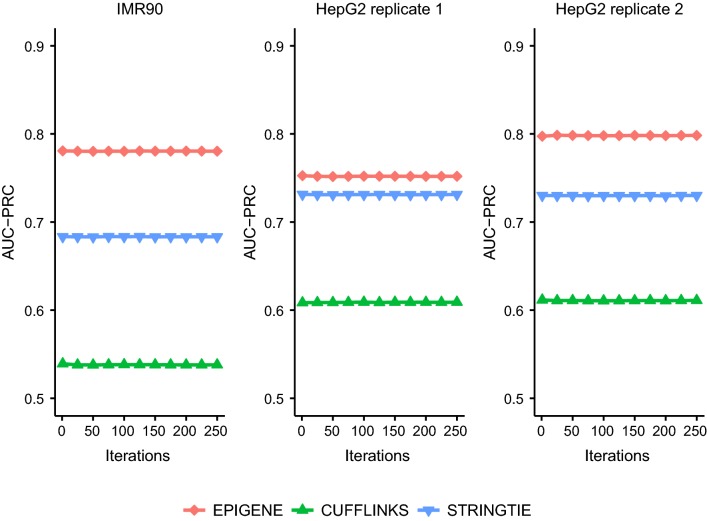
Performance of K562-trained EPIGENE models, Cufflinks and StringTie across cell lines

We applied the K562-trained EPIGENE model to IMR90 and HepG2 datasets and compared the predictions with Cufflinks and StringTie. The ChIP-seq profiles of RNA Polymerase II and GRO-seq profiles were used as performance indicator for both cell lines (see “Binarization of Nascent RNA-seq profiles” and “Performance evaluation” sections). As shown in Fig. 5 and Additional file 1: Figure S2, the K562-trained EPIGENE model consistently reports a higher AUC (PRC: 0.78, ROC: 0.77 in IMR90; PRC: 0.75, ROC: 0.77 in HepG2 replicate 1; PRC: 0.80, ROC: 0.80 in HepG2 replicate 2) compared to Cufflinks (PRC: 0.54, ROC: 0.54 in IMR90; PRC: 0.61, ROC: 0.64 in HepG2 replicate 1; PRC: 0.61, ROC: 0.64 in HepG2 replicate 2) and StringTie (PRC: 0.68, ROC: 0.72 in IMR90; PRC: 0.73, ROC: 0.77 in HepG2 replicate 1; PRC: 0.73, ROC: 0.78 in HepG2 replicate 2). These results suggest that EPIGENE generates accurate predictions across different cell lines, outperforming RNA-seq-based methods.

### Comparison with chromatin segmentation approaches

Currently several chromatin segmentation approaches (like ChromHMM and Segway) exist that provide chromatin state annotation using histone modifications. These approaches were inherently designed to provide a whole-genome chromatin state annotation and hence, the model parameters do not represent a specific topology. We examined the results of these approaches to evaluate their accuracy in identifying TUs.

We compared EPIGENE predictions with a widely used chromatin segmentation approach, ChromHMM, as both methods use a binning scheme. We did not include Segway in this comparison because it operates at single base pair resolution and, therefore restricts fair comparison of different profiles. Additionally, Segway is quite slower than chromHMM.

**Fig. 6:**
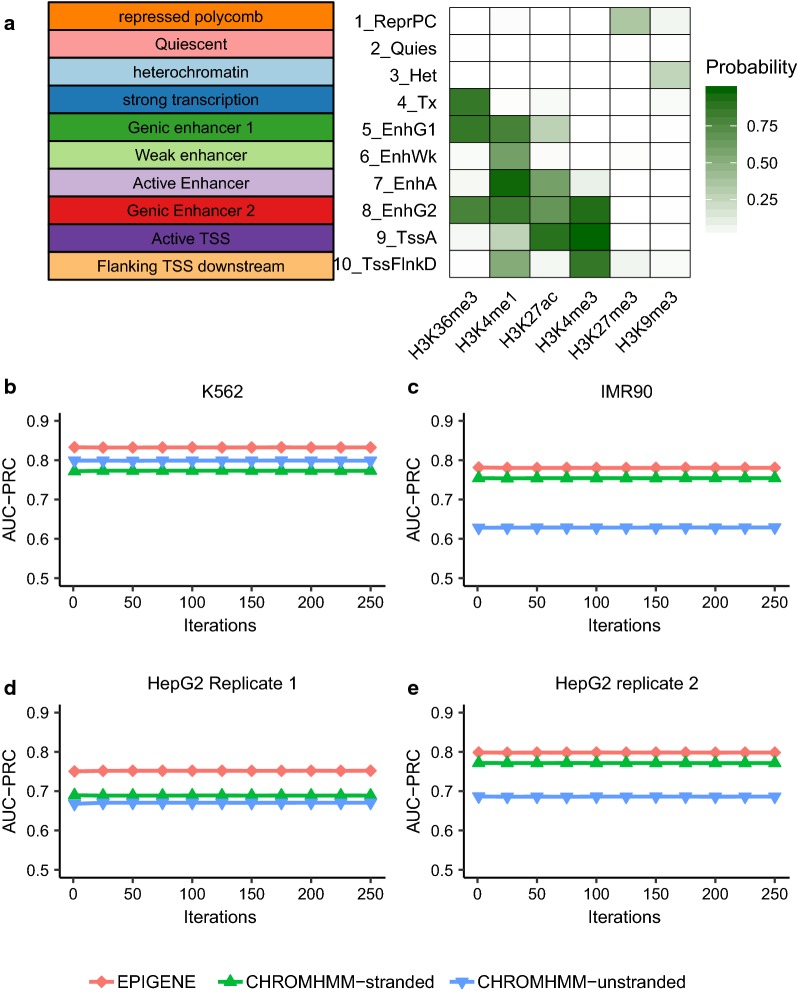
**a** Emission probabilities of ChromHMM model trained in K562 cell line. **b**–**e** Performance of K562-trained EPIGENE model and K562-trained ChromHMM model in K562, IMR90 and HepG2

TU identification with chromHMM was performed in two modes: strand-specific and unstranded. Strand-specific TUs were obtained by linking the promoter and transcription elongation states. We defined TU as a genomic region that begins with promoter state and proceeds through transcription elongation states. A promoter state was defined by an enrichment of H3K4me3 and H3K27ac (state 9 in Fig. 6a) and an elongation state was defined by an enrichment of H3K36me3 (state 4, 5 and 8 in Fig. 6a). Unstranded TUs were obtained by filtering chromHMM segmentations for transcription elongation states (state 4, 5 and 8 in Fig. 6a). The comparison was performed using the gold standard regions defined in “Comparison with RNA-seq based approaches” section. As shown in Fig. 6b–e and Additional file 1: Figure S3, EPIGENE consistently performed better (K562; ROC: 0.85, PRC: 0.83) than chromHMM strand-specific (K562, ROC: 0.73, PRC: 0.77) and unstranded TUs (K562, ROC: 0.79, PRC: 0.80). The lower AUC of strand-specific and unstranded chromHMM TUs was due to the presence of intronic enhancers and intermediate low coverage regions that resulted in fewer strand-specific chromHMM TUs and shorter strand-specific and unstranded chromHMM TUs (see Additional file 1: Figure S4).

### EPIGENE identifies transcription units with negligible RNA-seq evidence

Previous analyses (see “Histone modifications and RNA Polymerase II occupancy” and “Comparison with RNA-seq based approaches” sections) indicated the presence of TUs supported by RNA Polymerase II evidence but with reduced or no RNA-seq evidence. Here, we evaluated these TUs within and across cell lines by: (a) identifying cell type-specific TUs that showed TU characteristics but lack RNA-seq evidence, and (b) analysing the presence of microRNAs that were not identified by RNA-seq.

#### EPIGENE identifies cell type-specific transcription units

**Fig. 7:**
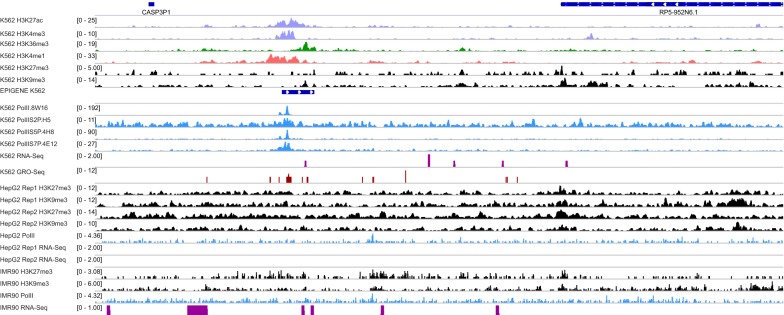
Example of EPIGENE-predicted TU that lacks RNA-seq evidence. The TU was predicted to be active in K562 but not in HepG2 and IMR90, and is located between pseudogene CASP3P1 and lncRNA RP5-952N6.1. The TU (shown in dark blue in EPIGENE-K562 track) shows an enrichment of H3K27ac and H3K4me3 at TSS (tracks shown in light violet), H3K36me3 in gene body (tracks shown in green), enhancer mark H3K4me1 few bps upstream of TSS (tracks shown in pink), GRO-seq in TSS (tracks shown in brown), K562 RNA Polymerase II in TSS and gene body (tracks shown in blue). The TU also shows an absence of repression marks H3K27me3 and H3K9me3 in K562 (tracks shown in black). We additionally observe the enrichment of repression mark in H3K27me3 in HepG2 and IMR90 indicating that the region is repressed in both these cell lines. There is a negligible RNA-seq evidence (shown in dark pink in K562-RNA-seq track) for this predicted TU

We created a consensus set of TUs by overlaying the EPIGENE predictions for K562, HepG2 and IMR90. This consensus TU set comprised 18,248 TUs, of which ~ 78% showed an enrichment for RNA Polymerase II. We identified 10,233 differential TUs of which 8047 were exclusive to cell lines (K562: 4247, IMR90: 2545, HepG2: 1255; see Additional file 1: Figure S5). We additionally identified 43 high-confidence cell-specific TUs (K562: 24, IMR90: 17, HepG2: 2; additional details in Additional file 3: Table S6), that lacked RNA-seq evidence but had typical characteristics of a TU, with RNA Polymerase II and GRO-seq enrichment at TSS and transcribed regions, H3K4me3 and H3K27ac enrichment at the TSS, and H3K36me3 enrichment in gene body (Fig. 7).

#### Identifying microRNAs that lack RNA-seq evidence

**Fig. 8:**
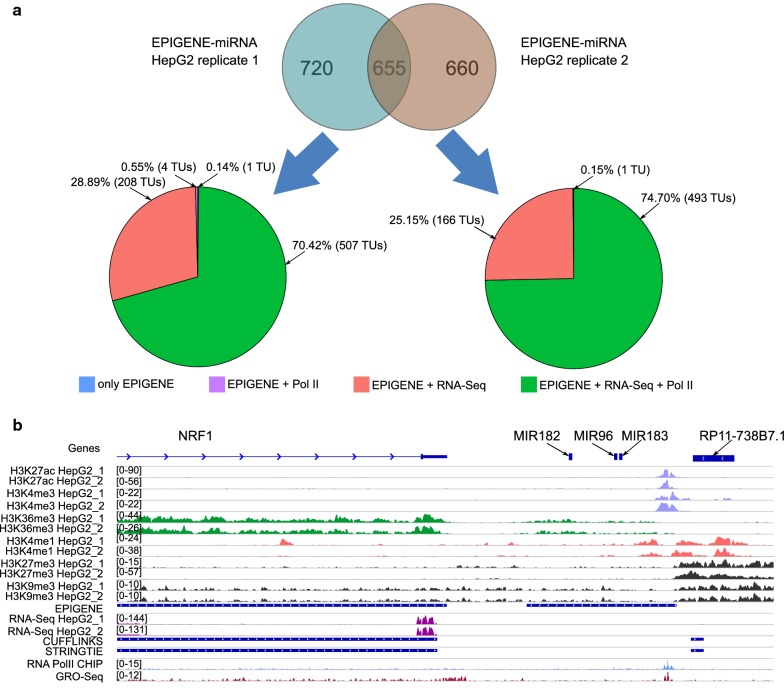
**a** Overview of potential primary miRNAs predicted by EPIGENE in HepG2. **b** Example of an EPIGENE-predicted TU overlapping a microRNA cluster in HepG2 cell line. This region is located between lincRNA RP11-738B7.1 and gene NRF1. The TU shows an enrichment of H3K27ac and H3K4me3 at TSS (tracks shown in light violet), H3K36me3 in gene body (tracks shown in green), enhancer mark H3K4me1 few bps upstream and downstream of TSS (tracks shown in pink), GRO-seq in TSS (tracks shown in brown) and RNA Polymerase II ChIP-seq in TSS (tracks shown in blue). The predictions also show an absence of repression marks H3K27me3 and H3K9me3 (tracks shown in black) and RNA-seq evidence (tracks shown in dark pink)

MicroRNAs are small (~ 22 bp), evolutionally conserved, non-coding RNAs [52, 53] derived from large primary microRNAs (pri-miRNA), that are processed to ~ 70 bp precursors (pre-miRNA) and consequently to their mature form by endonucleases [54, 55]. They regulate various fundamental biological processes such as development, differentiation, or apoptosis by means of post-transcriptional regulation of target genes via gene silencing [56, 57] and are involved in human diseases [58]. Due to the unstable nature of primary microRNA, traditional identification approaches relying on RNA-seq are challenging. Here, we investigated the presence of primary microRNAs that lack RNA-seq evidence across cell lines. We created a consensus TU set for individual cell lines (K562, HepG2 and IMR90) by combining EPIGENE and RNA-seq-based predictions. The RNA-seq-based predictions were obtained from Cufflinks and StringTie. The consensus TU set was overlapped with miRbase annotations [59] to obtain potential primary microRNA TUs. We identified 655 EPIGENE TUs in HepG2 (5% of total EPIGENE TUs common in both HepG2 replicates) that could be explained by miRbase annotations. We observed that majority of these were supported by RNA-seq and Polymerase II evidence (Fig. 8a and Additional file 1: Figure S6). We additionally identified 2 primary microRNA TUs in HepG2 cell line, which showed an enrichment for H3K27ac and H3K4me3 at their promoters, H3K36me3 in their gene body, and RNA Polymerase II in TSS and transcribed regions while lacking RNA-seq evidence. One of these TUs overlapped with a microRNA cluster located between RP-11738B7.1 (lincRNA) and NRF1 gene (Fig. 8b). This microRNA cluster has been shown to arise from the same primary miRNA and is also known to promote cell proliferation in HepG2 cell line [60, 61].

### Discussion

In this work, we introduced EPIGENE, a semi-supervised HMM that identifies active TUs using histone modifications. EPIGENE has TU (forward and reverse) and background sub-models. The TU sub-models were trained in a supervised manner on predefined training sets, whereas the background was trained in an unsupervised manner. This semi-supervised approach captures the biological topology of active TUs as well as the probability of occurrence of histone modifications in different parts of a TU.

We first showed that majority of the predicted TUs can be explained by existing gene annotations and were supported by RNA Polymerase II evidence. A quantitative comparison with RNA-seq revealed the presence of TUs with RNA Polymerase II enrichment but negligible RNA-seq evidence. Considering RNA Polymerase II ChIP-seq and Nascent RNA-seq as true transcription indicator, we compared the performance of EPIGENE with chromatin segmentation approach chromHMM and two RNA-seq-based approaches Cufflinks and StringTie. Based solely on the AUC of PRC and ROC curve as performance measure, EPIGENE achieves a superior performance than chromatin segmentation and RNA-seq-based approaches. We further showed that EPIGENE can be reliably applied across different cell lines without the need for re-training the TU states and accomplishes a superior performance than RNA-seq-based approaches.

We examined other performance scores like precision, sensitivity, and specificity values, and observed that the low AUC of RNA-seq-based approaches is due to RNA-seq mapping artefacts that resulted in higher number of false positives in Cufflinks and StringTie. We further evaluated the presence of differentially identified TUs in K562, HepG2, and IMR90 cell lines that lack RNA-seq evidence. The results suggested the presence of cell line-specific transcripts that lack RNA-seq evidence. We additionally identified potential microRNA precursors that lacked RNA-seq evidence presumably due to their instability. All of the aforementioned TUs showed an enrichment of RNA Polymerase II in TSS and gene body indicating that they had been transcribed.

It is important to note that EPIGENE does not differentiate between functional and non-functional units of a TU (exons and introns) as the association between histone modifications and alternative splicing is yet to be elucidated [62]. However, EPIGENE identifies active TUs with high precision as shown in “Comparison with RNA-seq based approaches” section and in the example regions presented in this work.

EPIGENE uses six core histone modifications that are available for many cell lines and species, which leads to a broad applicability. All the core histone modifications are essential for accurate TU identification, as the accuracy of TU prediction decreases in the absence of any of the core histone modification. In the absence of a core histone modification, imputation techniques such as ChromImpute [63] and PREDICTD [64] can be used to impute the missing histone modifications at 200-bp resolution and then use the imputed histone modification together with the available histone modifications to obtain active TUs. The accuracy of EPIGENE predictions also depends on the sequencing depth of the input histone modifications, therefore, high-quality ChIP-seq profiles of histone modifications would result in high confident TU annotation.

In summary, the superior performance within and across cell lines, identification of TUs, especially primary microRNAs lacking RNA-seq evidence as well as interpretability makes EPIGENE a powerful tool for epigenome-based gene annotation.

## Conclusion

With increasing efforts in the direction of epigenetics, many consortia continue to provide high-quality genome-wide maps of histone modifications, but determining the genome-wide transcriptomic landscape using this data has remained unexplored so far. Extensive evaluations in this work demonstrated the superior accuracy of EPIGENE over existing transcript annotation methods based on true transcription indicators. EPIGENE framework is user-friendly and can be executed by solely providing binarized enrichments for ChIP-seq experiments, without the need to re-train the model parameters. The resulting TU annotations agree with RNA Polymerase II ChIP-seq and Nascent RNA-seq evidence and can be used to provide a cell type-specific epigenome-based gene annotation.

## Materials and methods

### Library preparation of histone modifications ChIP-seq

For K562 cell line presented in this study, ChIP against six core histone modifications, H3K27ac, H3K27me3, H3K4me1, H3K4me3, H3K36me3 and H3K9me3, was performed. The sheared chromatin without antibody (input) served as control. 10 × 10^6^ K562 cells were cultured as recommended by ATCC. Chromatin immunoprecipitations were performed using the Diagenode auto histone ChIP-seq kit and libraries were made using microplex kits according to manufacturer’s instructions and 10 PCR cycles.

### Library preparation of RNA Polymerase II ChIP-seq

K562 cells were cultured in IMDM (#21980Gibco) with 10% FBS and P/S. Cells at a concentration of 1.2 mio/ml were fixed with 1% formalin at 37 °C for 8 min. Nuclei were isolated with a douncer, chromatin concentration was measured and 750 µg chromatin per CHIP was used. Samples were sonicated with Biorupter for 33 cycles (3 × 11 cycles). Chromatin, antibodies (RNA Pol II Ser2P (H5), RNA Pol II Ser5P (4H8), RNA Pol II Ser7P (4E12) and PolII (8WG16)) and protein G beads were combined and rotated at 4 °C. For elution 250 µl elution buffer (1% SDS) was used and after reverse crosslinking DNA was isolated by phenol chloroform extraction and eluted in 1xTE. Final concentration was measured by Qubit. Bioanalyzer was done to check fragment sizes.

### Sequencing and processing of ChIP-seq data

Sequencing for RNA Polymerase II and histone modifications was performed on an Illumina Highseq 2500 using a paired end 50-flow cell and version 3 chemistry. The resulting raw sequencing reads were aligned to the genome assembly “hs37d5” with STAR [65] and duplicates were marked using Picard tools [66]. We used *plotFingerprint* which is a part of deepTools [67] to access the quality metrics for all ChIP-seq experiments.

### Processing of RNA-seq data

The raw reads from RNA-seq experiments were downloaded from European Nucleotide Archive (SRR315336, SRR315337 for K562), European Genome Archive (EGAD00001002527 for HepG2) and ENCODE (ENCSR00CTQ for IMR90) and were aligned to the genome assembly “hs37d5” with STAR [65].

### Processing of Nascent RNA-seq data

The transcript annotation for K562 obtained from TT-seq were downloaded from Gene Expression Omnibus (GEO) (GSE75792). The genomic co-ordinates of transcripts were lifted over to hg19. For HepG2, raw reads from GRO-seq were downloaded from GEO (GSM2428726). The raw reads were aligned to the hg19 and the pre-processing was done based on the instructions specified in Liivard et al. [51]. For IMR90, we used GRO-seq profiles generated in Jin et al. [49]. The profiles were downloaded from GEO (GSM1055806) and lifted over to hg19.

### Binarization of ChIP-seq profiles

EPIGENE requires the enrichment values of IHEC class 1 histone modifications in a binarized data form or a “class matrix” to learn a transcription state model. This was done by partitioning the mappable regions of the genome of interest into non-overlapping sub-regions of the same size called bins. In the current setup, the transcription states are analysed at 200-bp resolution, as it roughly corresponds to the size of a nucleosome and spacer region. Given the ChIP and input alignment files for each of the histone modifications, the class matrix for multivariate HMM was generated using the following approach:

1. *Obtaining read counts* Read counts for all the bins was computed using *bamCount* method from R package *bamsignals* [68], with the following parameter settings: mapqual = 255, filteredFlag = 1024, paired.end = midpoint.
2. *Enrichment calling and binarization* After having obtained the read counts, binarization of ChIP-seq signal for the histone modifications and RNA Polymerase II across all bins $E\left( {{\text{bin}},{\text{HM}}} \right)$ and $E\left( {{\text{bin}},{\text{RNAPolIIChIP}}} \right)$ were computed using *enrichR* (binFilter = zero) and *getClasses* (fdr = 0.2) method from *normR* [44]. This step yields the class matrix that serves as an input for the multivariate HMM.

### The EPIGENE model

EPIGENE uses a multivariate HMM (shown in Fig. 1a (ii)) to model the class matrix and identify active transcription units. Class matrix $C$ is a *m* × *n* matrix, where $m =$ total number of 200 bp bins, and, $n$ = number of histone modifications. Each entry $Cij$ in the class matrix $C$ corresponds to the binarized enrichment in *i*th bin for the *j*th histone modification. The model constitutes $k$ number of hidden states (which is an input parameter of the algorithm), and each row of the class matrix corresponds to a hidden state. The emission probability vector for each hidden state corresponds to the probability with which each histone mark was found for that hidden state. The transition probabilities between the states enable the model to capture the position biases of gene states relative to each other. The emission probabilities of each state represent the probability with which each histone mark occurs in a state. Given this model, the algorithm does the following:

1. Initializes the emission, transition, and initial probabilities.
2. Fits the emission, transition, and initial probabilities using the Baum–Welch algorithm [69].
3. As we are concerned about the most probable sequence of active transcription unit, therefore the sequence of hidden states was inferred using the Viterbi algorithm [70].

### Training the model parameters

The transition and emission probabilities of the multivariate HMM were trained using GENCODE annotations with the following approach:

1. Bins overlapping gencode transcripts were identified and termed as gencode bins.
2. The gencode bins were categorized as TSS, TTS, 1st, internal and last exon and intron bins, and were subsequently grouped based on transcript IDs.
3. The coverage (in bp) of individual transcription unit component (i.e. TSS, 1st exon, 1st intron, etc.) for each transcript was computed to generate the coverage list, where each entry of the coverage list contains the coverage information (in bp) for individual transcripts.
4. The transition probability of each “transcription unit state” was computed from the coverage list, and the missing probabilities from and to the “background state” were generated in an unsupervised manner.
5. The gencode transcripts were filtered to obtain transcripts that report an enrichment for RNA Polymerase II. This was done by clustering the binarized enrichment values of RNA Polymerase II in TSS and TTS bins of the transcripts and obtaining TSS and TTS bins that report a high cluster mean for RNA Polymerase II. The emission probability of each “transcription unit state” was computed from class matrix and coverage of these transcripts (coverage computed from Step 2). The missing emission probabilities for the background states were trained in an unsupervised manner.

### Binarization of Nascent RNA-seq profiles

Nascent RNA transcript annotation for GRO-seq profiles was obtained using groHMM [71]. For HepG2, transcript annotation was obtained from GRO-seq using default parameter values, while for IMR90, transcript annotation was obtained from GRO-seq using parameter values specified in Chen et al. [71]. In K562, transcript annotation was obtained from Schwalb et al. [17]. For a given cell line ${\text{C}}$, the presence/absence of Nascent RNA-seq profiles across 200 bp bins $E_{C} \left( {{\text{bin}},{\text{NascentRNA}}} \right)$ is given by:

$$E_{\text{C}} \left( {{\text{bin}},{\text{NascentRNA}}} \right) = \left\{ {\begin{array}{*{20}c} 1 \\ 0 \\ \end{array} } \right.\begin{array}{ll} \quad {{\text{if}}\;O\left( {{\text{bin}},{\text{Tr}}_{\text{C}} } \right) \ge 1} \\ \quad {\text{otherwise}} \\ \end{array} ,$$

where $O\left( {{\text{bin}},{\text{Tr}}_{\text{C}} } \right)$ is the overlap between the bin and cell line ${\text{C}}$ nascent RNA transcripts ${\text{Tr}}_{\text{C}}$.

### Performance evaluation

The performance of EPIGENE and RNA-seq-based transcript prediction approaches was evaluated using RNA Polymerase as performance indicator. This was done by removing assembly gaps in the genomic regions of interest and partitioning the remaining contigs into non-overlapping bins of 200 bps. The actual transcription status of each 200 bp bin was given by the observed binarized RNA Polymerase II ChIP-seq and Nascent RNA-seq enrichment in the bin. The actual transcription ${\text{AT}} \left( {\text{bin}} \right)$ was given by:

$${\text{AT}} \left( {\text{bin}} \right) = \left\{ {\begin{array}{*{20}c} 1 \\ 0 \\ \end{array} } \right.\begin{array}{ll} \quad {{\text{if}}\;E\left( {{\text{bin}},{\text{RNAPolIIChIP}}} \right) \cap E\left( {{\text{bin}},{\text{NascentRNA}}} \right) = 1} \\ \quad {\text{otherwise}} \\ \end{array} ,$$

where $E\left( {{\text{bin}},{\text{RNAPolIIChIP}}} \right)$ is enrichment of RNA Polymerase II ChIP-seq (obtained from “Binarization of ChIP-seq profiles” section) and $E\left( {{\text{bin}},{\text{NascentRNA}}} \right)$ is enrichment of Nascent RNA-seq in the bin (obtained from “Binarization of Nascent RNA-seq profiles” section).

The predicted transcription status of the bin for method m, ${\text{PT}}_{\text{m}} \left( {\text{bin}} \right)$ was given by:

$${\text{PT}}_{\text{m}} \left( {\text{bin}} \right) = \left\{ {\begin{array}{ll }1 \\ 0 \\ \end{array} } \right.\begin{array}{ll} \quad {{\text{if}}\;O\left( {{\text{bin}},P_{\text{m}} } \right) \ge 1} \\ \quad {\text{otherwise}} \\ \end{array} ,$$

where $O\left( {{\text{bin}},P_{\text{m}} } \right)$ is the overlap between the bin and method m predictions $P_{\text{m}}$.

The predictions of EPIGENE and other RNA-seq-based approaches were evaluated by computing the area under curve for precision–recall (AUC-PRC) and receiver-operating characteristic curve (AUC-ROC) with primary focus on AUC-PRC. Considering a very high class imbalance, i.e. $${\text{bins}}_{{{\text{RNAPolymeraseII}}^{ + } }}$$$$\ll {\text{bins}}_{{{\text{RNAPolymeraseII}}^{ - } }}$$, the AUC-PRC and AUC-ROC are computed using random sampling as:

$${\text{AUC}} = {\text{mean}}\left( {L_{\text{AUC}} } \right) - \left( {\frac{{{\text{stdDev}}\left( {L_{\text{AUC}} } \right)}}{\sqrt n }} \right),$$

where $n$ is the sampling size or number of iterations and $L_{\text{AUC}}$ is the list of AUCs obtained for sampling size $n$.

## Supplementary information

**Additional file 1.** Data details and additional results. Details of datasets used and additional results.

**Additional file 2.** RNA Polymerase II enrichment. RNA Polymerase II enrichment in consensus TU set.

**Additional file 3.** Cell specific TUs. Additional details about cell specific TUs that lack RNA-seq evidence.

## Data Availability

Data for ChIP-seq experiments for K562 cell line are available via European Nucleotide Archive (PRJEB34999). Additional details about other ChIP-seq and RNA-seq data used in this work can be found in Additional file 1: Table S1. EPIGENE code is available at: https://github.com/imbbLab/EPIGENE.
