# Supplementary material for: EPIGENE: genome-wide transcription unit annotation using a multivariate probabilistic model of histone modifications
Source: Epigenetics Chromatin. 2020 Apr 7;13:20. doi: 10.1186/s13072-020-00341-z (PMC7137282; doi:10.1186/s13072-020-00341-z)
Supplement: Supplementary file 1 — Additional file 1. Data details and additional results. Details of datasets used and additional results. [file 13072_2020_341_MOESM1_ESM.pdf]

1. Data

Table 1 lists the datasets used in this study. The RNA-Seq and ChIP-seq data were processed as mentioned in Section 4.3. The sequencing reads were binned in 200 bp bins and the raw ChIP read counts were normalized and binarized using normR. In this study we consider only uniquely mapped reads.

Cell line	Residue	Sequencing	Accession number	Source
IMR90	H3K27ac	ChIP-seq	ENCSR002YRE	ENCODE
IMR90	H3K4me3	ChIP-seq	ENCSR087PFU	ENCODE
IMR90	H3K4me1	ChIP-seq	ENCSR831JSP	ENCODE
IMR90	H3K36me3	ChIP-seq	ENCSR437ORF	ENCODE
IMR90	H3K27me3	ChIP-seq	ENCSR431UUY	ENCODE
IMR90	H3K9me3	ChIP-seq	ENCSR055ZZY	ENCODE
IMR90	input	ChIP-seq	ENCSR001BSB, ENCSR704GTT	ENCODE
IMR90	RNA Polymerase II-Input	ChIP-seq	ENCSR000EFL	ENCODE
IMR90	RNA Polymerase II	ChIP-seq	ENCSR000EFK	ENCODE
IMR90	mRNA	RNA-Seq	ENCSR000CTQ	ENCODE
HepG2	RNA Polymerase II-Input	ChIP-seq	ENCSR000EEM	ENCODE
HepG2	RNA Polymerase II	ChIP-seq	ENCSR000EEN	ENCODE
HepG2	H3K27ac, H3K4me3, H3K4me1, H3K36me3, H3K27me3, H3K9me3, Histone mark input, RNA-Seq	ChIP-seq	EGAD00001002527	DEEP
K562	mRNA	RNA-Seq	SRR315336, SRR315337	European nucleotide archive
K562	Nascent RNA	TT-Seq	GSE75792	Gene Expression Omnibus
K562	Nascent RNA	GRO-Seq	GSM1480325	Gene Expression Omnibus
HepG2	Nascent RNA	GRO-Seq	GSM2428726	Gene Expression Omnibus
IMR90	Nascent RNA	GRO-Seq	GSM1055806	Gene Expression Omnibus

Table S1: Experimental data used in this study

2. Summary statistics

The summary statistics of transcription units predicted by EPIGENE, STRINGTIE and CUFFLINKS can be seen in Table 2,3 and 4.

	genes	+ strand	- strand	median length
**all**	24,571	13,410	11,161	7,800
**gencode V19 + chess 2.1 same strand overlap**	18,184	9,774	8, 410	9,800
**gencode V19 + chess 2.1 any overlap**	23,542	12,921	10,621	8,400
**no match**	1,029	489	540	2,000

Table S2: Summary statistics of transcription units predicted by EPIGENE

	genes	+ strand	- strand	median length
**all**	101,656	50,636	51,020	5,481
**gencode V19 + chess 2.1 same strand overlap**	93,006	46,448	46,558	6,719
**gencode V19 + chess 2.1 any overlap**	97,300	48,531	48,769	6,110
**no match**	4,356	2,105	2,251	613

Table S3: Summary statistics of transcription units predicted by STRINGTIE

	genes	+ strand	- strand	median length
**all**	32,079	15,262	15,095	8,851
**gencode V19 + chess 2.1 same strand overlap**	26,452	12,986	12,671	16,486
**gencode V19 + chess 2.1 any overlap**	27,157	13,320	13,042	15,392
**no match**	4,992	1,942	2,053	962

Table S4: Summary statistics of transcription units predicted by CUFFLINKS

3. False positives due RNA-Seq mapping artefacts

We investigated the cause of higher AUC for EPIGENE compared to RNA-Seq based approaches and found that this due to slightly higher number of false positive resulting due to RNA-Seq mapping artefacts.

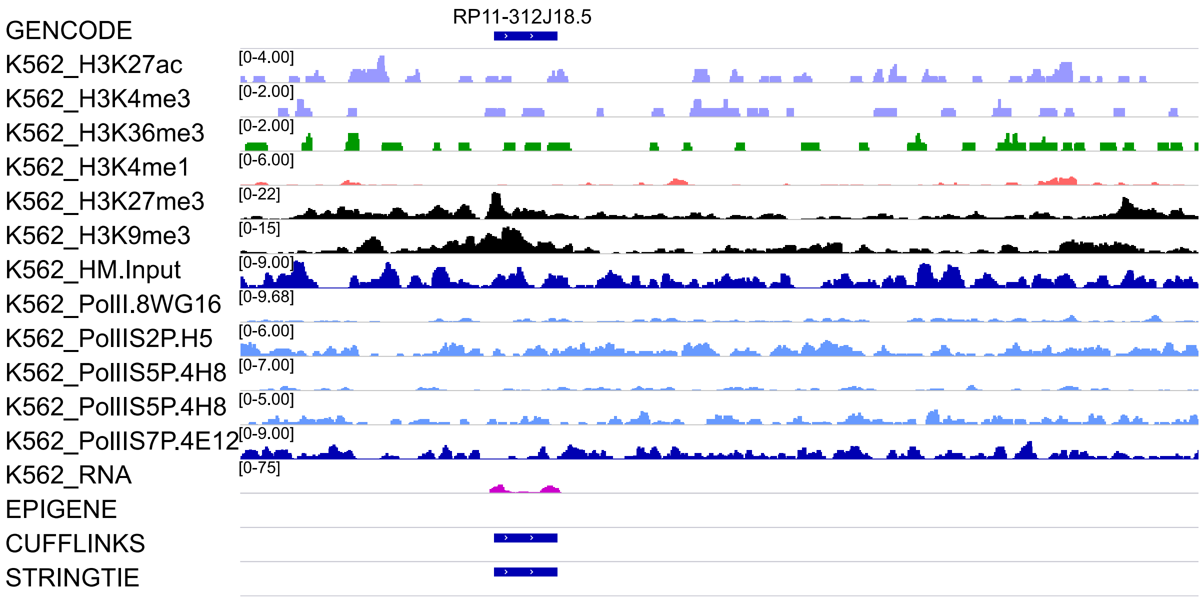

Figure S1: An example of CUFFLINKS and STRINGTIE due to spurious read mapping. This is a repetitive sequence occurring in chromosome 1,5,6,X, We observe an enrichment of repressive histone modifications like H3K27me3 and H3K9me3 (tracks shown in black) indicating that this is a repressive region.

4. Robustness of the EPIGENE model

The robustness of EPIGENE was examined by testing K562-trained models on two other cell lines: IMR90 and HepG2. The similar performance score of independent EPIGENE models reflected its robustness.

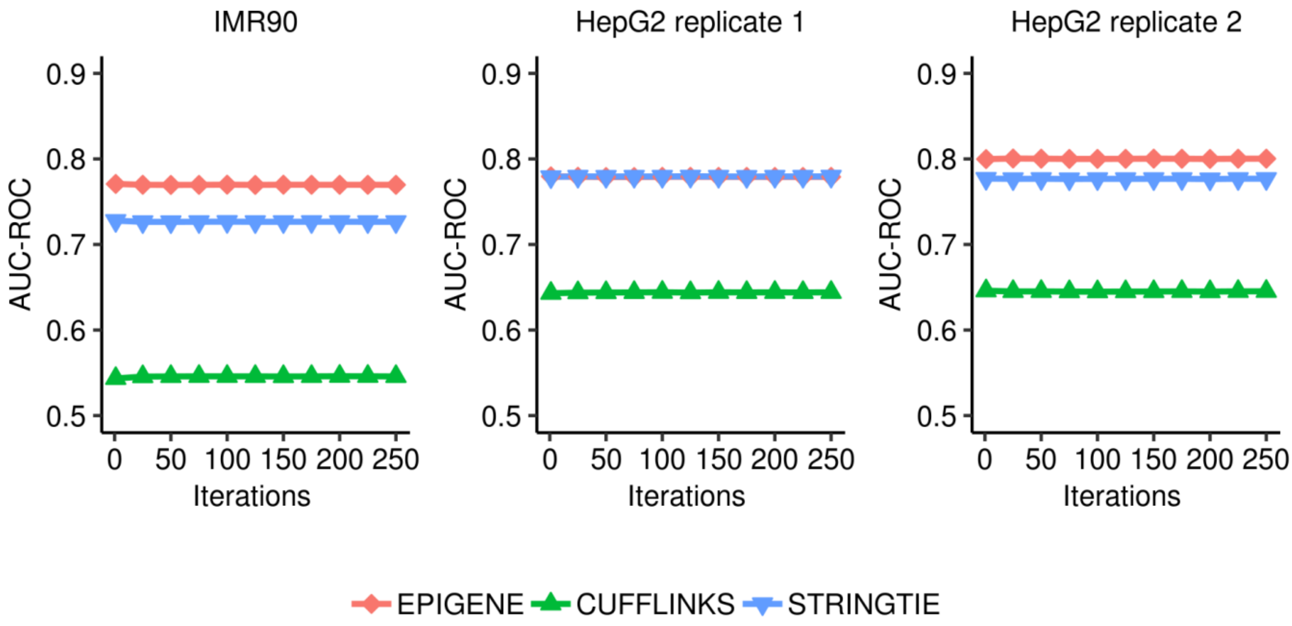

Figure S2: Comparison of K562 trained EPIGENE models with STRINGTIE and CUFFLINKS across 2 cell lines. ROC curves show that EPIGENE achieves a superior performance for 3 data sets compared to other approaches.

5. Comparison with existing chromatin segmentation approaches

We compared EPIGENE with the whole-genome chromatin state annotation approach ChromHMM as it uses the same binning scheme as EPIGENE. We compared the performance of K562-trained EPIGENE and ChromHMM across 3 cell lines. A superior performance of EPIGENE demonstrates benefits of incorporating topological information to the model parameters of a probabilistic model.

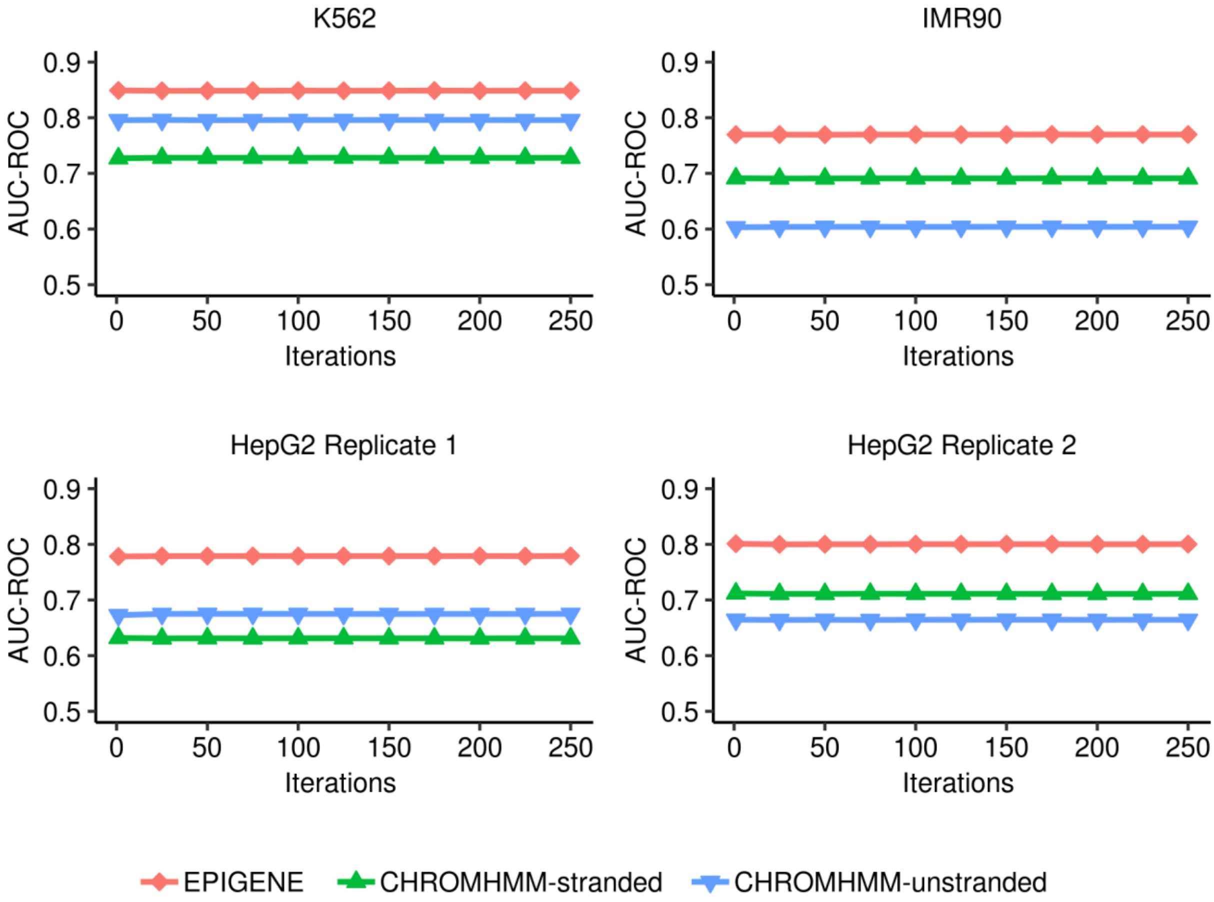

Figure S3: Comparison of K562 trained EPIGENE and ChromHMM models across 3 cell lines. ROC curves show that EPIGENE achieves a superior performance for 3 data sets compared to ChromHMM.

We further analysed the length of TUs predicted by EPIGENE and ChromHMM. A comparison of length distributions of EPIGENE and ChromHMM TUs revealed that ChromHMM result in

shorter TUs. Additionally, a strand specific TU identification using ChromHMM resulted in less number of TUs. This is due to the presence of intronic enhancers.

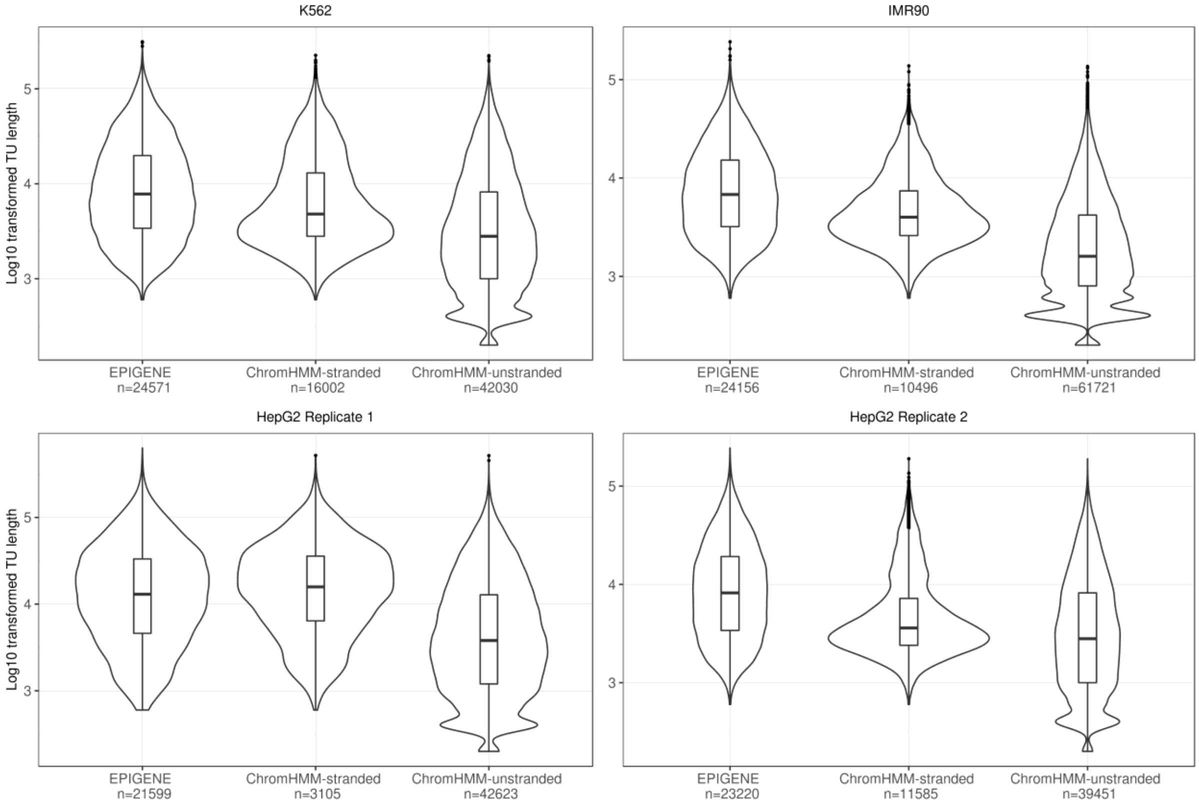

Figure S4: Comparison of log transformed TU length distributions of EPIGENE and ChromHMM TUs across 3 cell lines. The violin plots reveals that ChromHMM TUs are relatively shorter than EPIGENE TUs.

6. Distribution of EPIGENE predictions across cell lines

We create a consensus TU set using the approach to identify cell specific TUs.

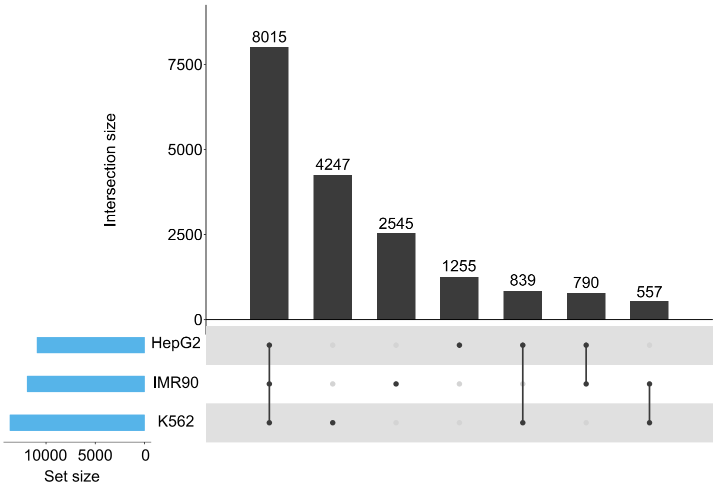

Figure S5: Distribution of EPIGENE TUs across cell lines

7. Summary statistics of EPIGENE TUs overlapping miRNAs

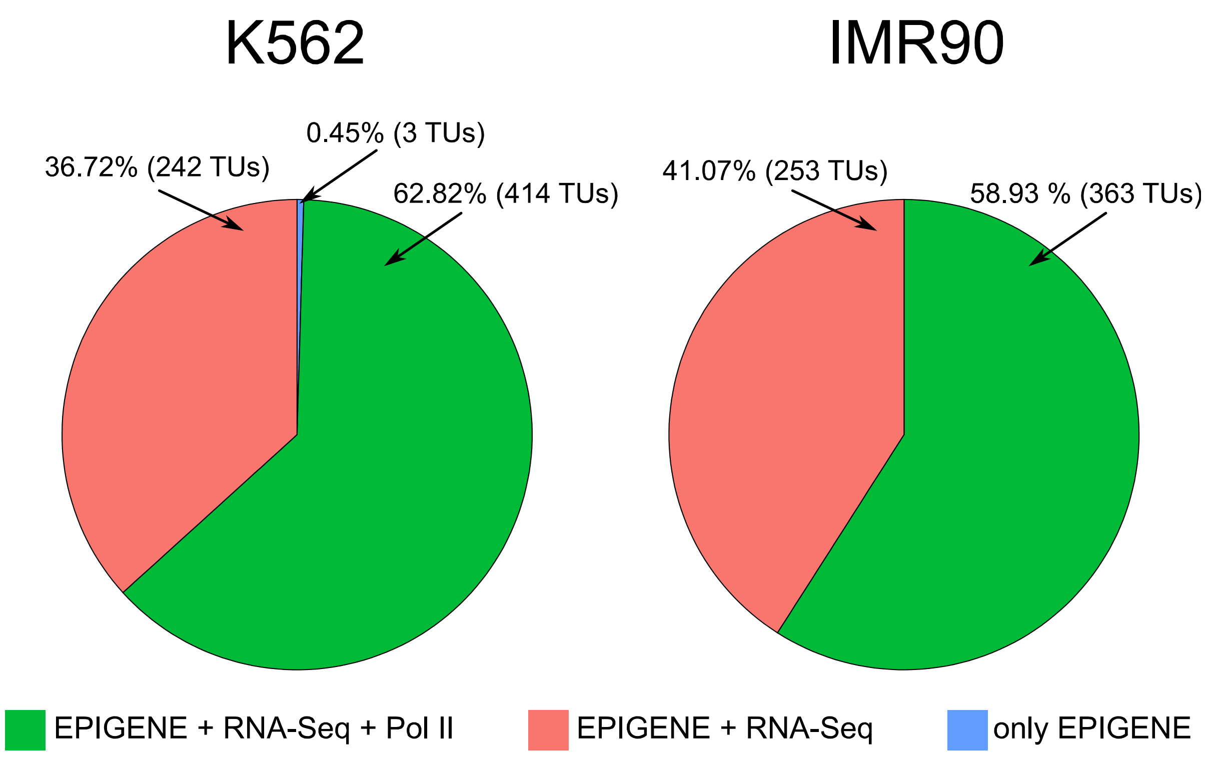

Figure S6: Majority of EPIGENE TUs overlapping miRNAs can be explained by RNA-Seq and Polymerase II evidence
